# PSO-Based Smart Grid Application for Sizing and Optimization of Hybrid Renewable Energy Systems

**DOI:** 10.1371/journal.pone.0159702

**Published:** 2016-08-11

**Authors:** Mohamed A. Mohamed, Ali M. Eltamaly, Abdulrahman I. Alolah

**Affiliations:** 1 Electrical Engineering Dept., King Saud University, Riyadh 11421, Saudi Arabia; 2 Electrical Engineering Dept., Minia University, Minia 61519, Egypt; 3 Sustainable Energy Technologies Center, King Saud University, Riyadh 11421, Saudi Arabia; 4 Electrical Engineering Dept., Mansoura University, Mansoura 35516, Egypt; Beihang University, CHINA

## Abstract

This paper introduces an optimal sizing algorithm for a hybrid renewable energy system using smart grid load management application based on the available generation. This algorithm aims to maximize the system energy production and meet the load demand with minimum cost and highest reliability. This system is formed by photovoltaic array, wind turbines, storage batteries, and diesel generator as a backup source of energy. Demand profile shaping as one of the smart grid applications is introduced in this paper using load shifting-based load priority. Particle swarm optimization is used in this algorithm to determine the optimum size of the system components. The results obtained from this algorithm are compared with those from the iterative optimization technique to assess the adequacy of the proposed algorithm. The study in this paper is performed in some of the remote areas in Saudi Arabia and can be expanded to any similar regions around the world. Numerous valuable results are extracted from this study that could help researchers and decision makers.

## 1. Introduction

In recent years, interest in renewable energy sources (RES) for power generation is progressively gaining significance in the entire world due to fossil fuel depletion, high cost, and increasing environmental concerns. Therefore, there is a big trend to use RES to address the power generation especially for the isolated or remote areas. Utilization of different RES with storage and backup units to form a hybrid renewable energy system (HRES) can give a more economic and reliable source of energy [1]. But, due to the non-linear response of system components and the random nature of RES and load profile, smart grid is utilized to suit and incorporate these units in order to move the power around the system as efficiently and economically as possible [2].

One of the most important issues in the recent studies is to optimize the components of HRES to meet the load requirements with possible minimum cost and highest reliability. In view of the complexity of optimization of the HRES, it was imperative to discover effective optimization methods ready to get accurate optimization results. Particle swarm optimization (PSO) algorithm is recommended as a standout amongst the most valuable and promising methods for optimizing the HRES because of using the global optimum to locate the best solution [3]. PSO algorithm is designed based on swarm intelligence and used to handle the complex optimization problems [4]. Like other population-based optimization algorithms, PSO begins with a random initialization of particles in the search space. Every particle is invested with a random position and a random velocity at the beginning, and then adjusts its search patterns in view of its own experience and experiences of other individuals [5].Owing to its simplicity, effectiveness and low computational cost; PSO has gained significant popularity and improvements [6]. In the last decade, numerous authors developed PSO to fulfill several HRES optimization objectives and constraints [7–15]. Boonbumroong et al. [7] utilized PSO to minimize the life-cycle cost of a stand-alone PV/wind/diesel system to feed a certain load. The optimization constraint was that the hourly energy demand must be satisfied by the amount of generated energy. A PSO algorithm was applied in [8–12] for optimum sizing of a hybrid energy system for supplying a certain load. The optimization objective was to minimize the system cost with the constraint of having specific reliability. The HRES includes PV/wind/battery as in [8, 10, 12], PV/wind/fuel cell in [9], and PV/wind/tidal/battery energy sources in [11]. Hakimi et al. [13] used PSO to minimize the total cost of a stand-alone hybrid energy system formed by wind units, electrolyzers, a reformer, an anaerobic reactor, fuel cells and some hydrogen tanks in order such that the demand is met. The optimization constraint was the stored energy in hydrogen tanks. An optimization problem using PSO to solve the PV/wind capacity coordination for a time-of-use rate industrial user was introduced in [14] with the aim of maximizing the economic benefits of investing in the wind and PV generation systems. The optimization constraint was that the generation from HRES must not to be greater than the maximum annual load. Wang et al. [15] used multi-objectives PSO algorithm to optimize a hybrid PV/wind/battery energy system on the basis of cost, reliability, and emission criteria without considering load management.

Demand response and demand profile improvement have been the center of consideration by numerous researchers [16–19]. Conejo et al. [16] carried out the demand response by adjusting the hourly load level in response to the hourly electricity prices. The authors in [17] proposed an optimum and automatic scheduling framework using linear programming optimization, to accomplish a trade-off between minimizing the electricity payment and minimizing the waiting time for the loads operation, in the presence of a real-time pricing tariff. Incentivizing consumers to achieve an ideal load profile suitable for utilities was one of the proposed solutions for demand profile improvement [18]. Barley et al. [19] accomplished sizing of the HRES components and control the generated energy price based on the trade-off between the system cost and the percent unmet load. The authors utilized Hybrid2 software in conjunction with a disentangled time-series model.

In summary, although PSO has been used in several studies and delivers promising results, most of these studies focus on one issue like sizing, emission control, reliability or cost only [7–14] and they didn’t address multi-objectives or multi-constraints analysis of HRES. Furthermore, all of the optimization approaches used in the above studies [7–19] didn’t take into account the smart grid applications like load shifting and management and relied on the consumer’s endeavors or encouragement consumers to improve the load profile or decrease peak demand, which makes it hard to be accomplished. Most of these approaches depend on the presence of a real-time pricing tariff for cost or peak demand reduction which is not an ideal solution. In addition, the above studies ignored the dummy energy and didn’t provide approaches to exploit it [7–19].

In this paper, an optimal sizing algorithm based on smart grid applications is introduced to determine the optimum size of stand-alone hybrid PV/wind/battery/diesel energy systems so as to meet the load requirements with minimum cost and highest reliability. Load shifting-based load priority is presented as one of smart grid applications by dividing the load into two factions, high priority load (HPL) and low priority load (LPL). HPL is due to essential activities and the appliances involved in these activities have fixed scheduling requirements. Therefore, HPL must be supplied whatever the generation conditions. LPL is due to flexible activities and the appliances involved in these activities can have flexible scheduling requirements. Therefore, LPL can be shifted and supplied from the surplus generation time of RES. Also, a proposed methodology for exploitation of the dummy energy is exhibited. Furthermore, a PSO algorithm is employed for seeking the optimum size of HRES and minimum cost of energy of the system under study. A comparison between the results obtained from PSO algorithm and those from the iterative optimization techniques (IOT) is introduced. IOT is a mathematical procedure that generates a sequence of improving approximate solutions for a class of problems. In order to find the optimal solution of a system, the IOT uses an initial guess to generate successive approximations to a solution. IOT is used to solve problems of nonlinear programming differ according to the objective functions and constraints. In some cases, the evaluation of the complex objective function using IOT requires a large computational effort and increases the computational time of each iteration. Parallel implementation of PSO (PIPSO) is a modern method utilized in this paper to distribute the evaluation of the fitness function and constraints among the ready-made processors or cores, and to speed up the optimization process. A comparison between utilizing PIPSO and utilizing a serial implementation of PSO (SIPSO) is presented.

## 2. Modeling of Hybrid Renewable Energy System

**Fig 1:**
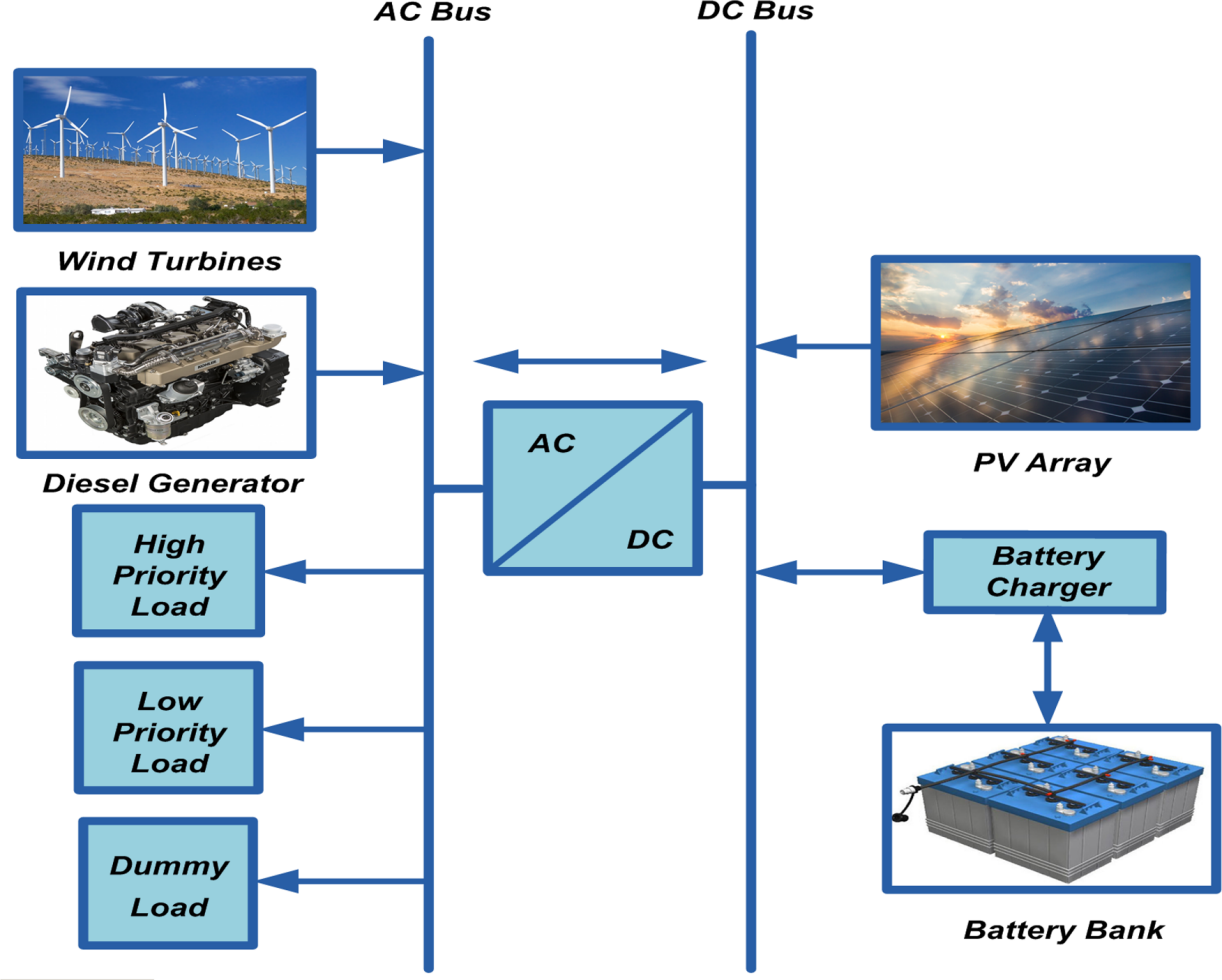
Schematic drawing of the proposed system.

The schematic drawing of the proposed stand-alone hybrid PV/wind/battery/diesel energy system is shown in Fig 1. As shown in this figure, wind turbines (WT) are connected to AC-bus, PV array is connected to DC-bus, and battery charger is connected to the DC-bus to charge the battery bank from the respective WT and PV array through a bi-directional AC/DC converter. Diesel generator (DG) is connected to AC-bus as a backup source of energy. Finally, a group of loads, HPL, LPL, and dummy load are connected to AC-bus as the load demand for the HRES.

A mathematical modeling of the proposed HRES parts is detailed in the following subsections:

### 2.1 Resources and Load Data

**Fig 2:**
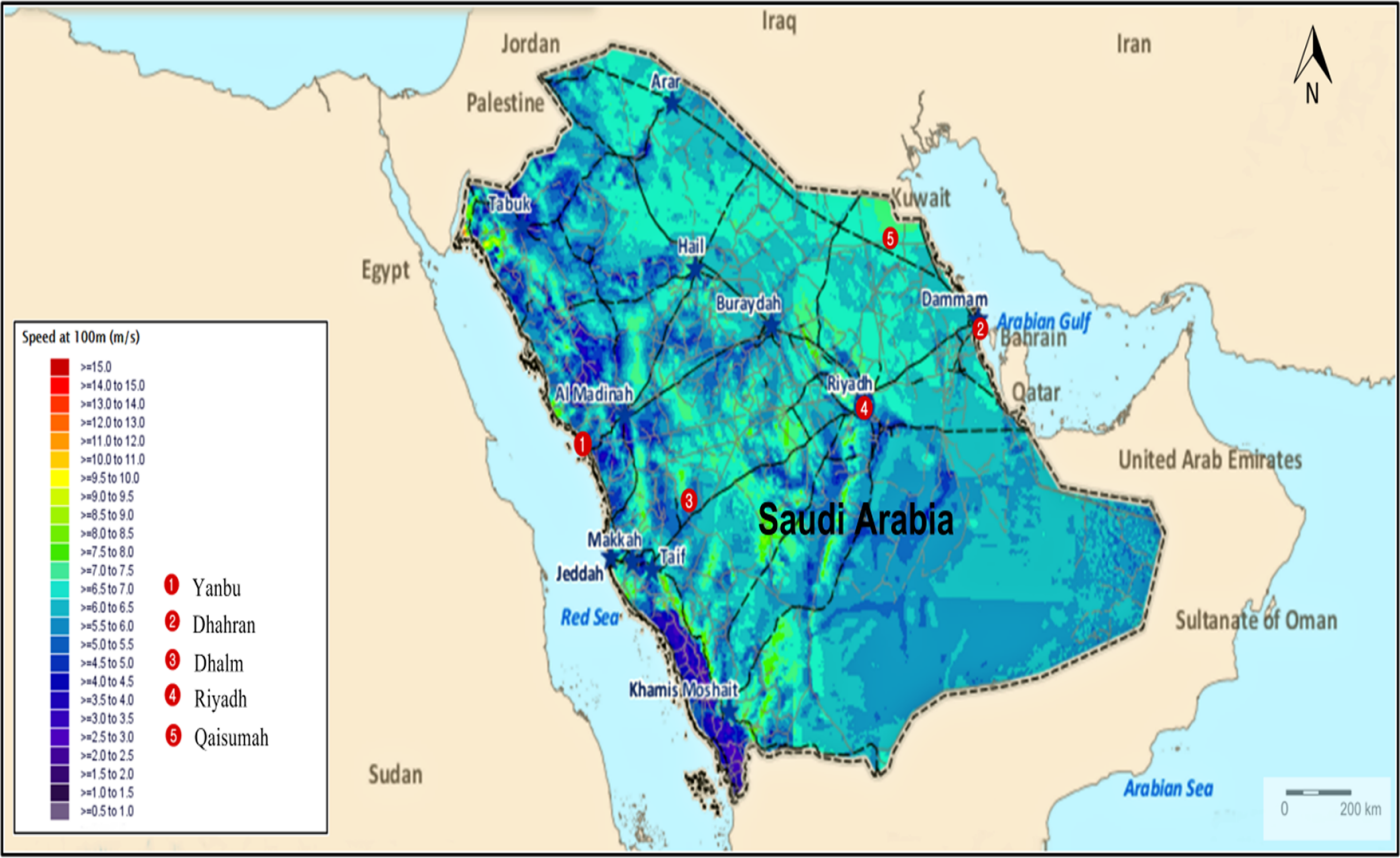
Wind speed in Saudi Arabia at the height of 100 m.

**Fig 3:**
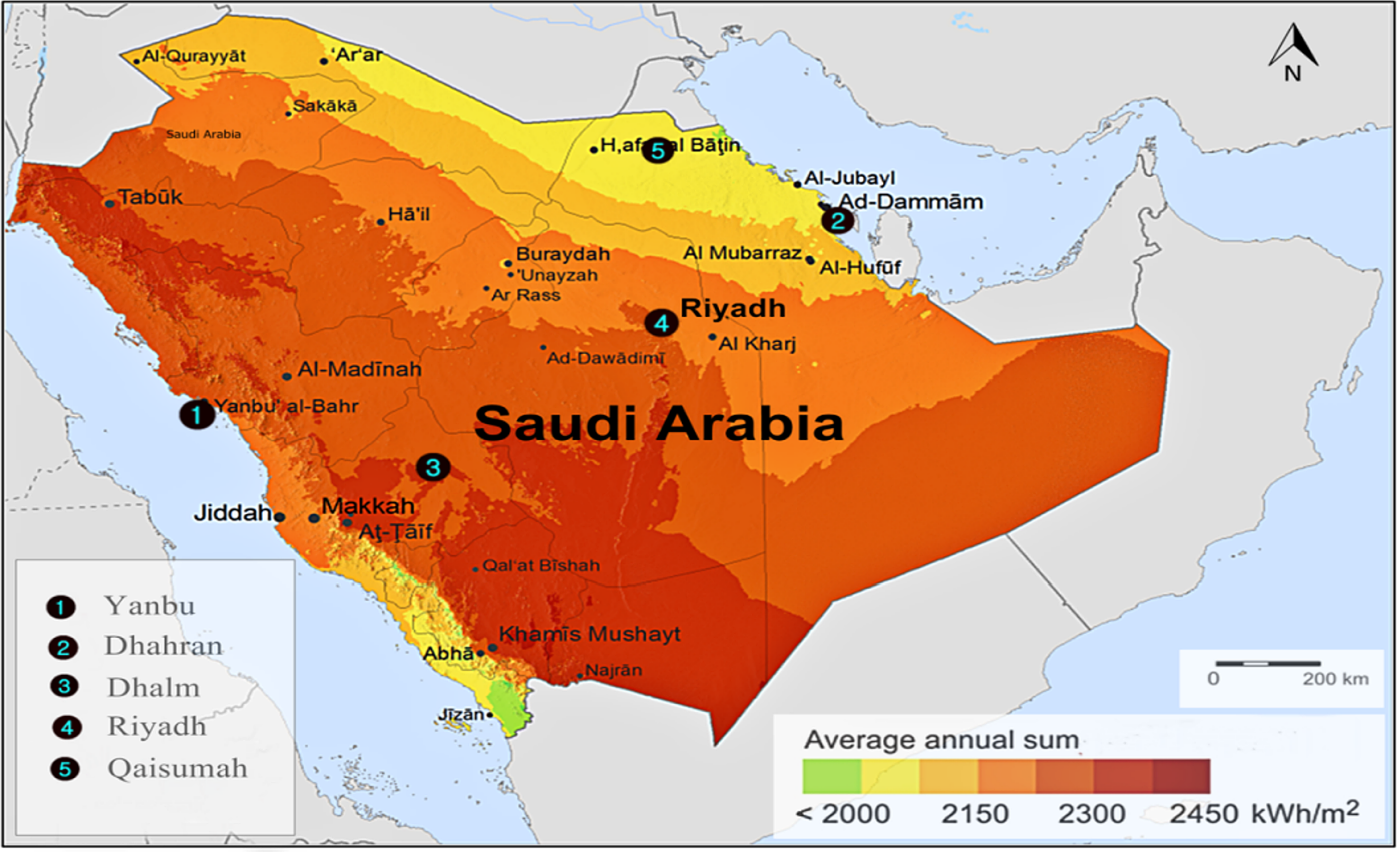
Global horizontal radiation in Saudi Arabia.

The hourly data of the wind speed, solar radiation, and temperature for five sites in Saudi Arabia are used as a case study. These sites are Yanbu, Dhahran, Dhalm, Riyadh, and Qaisumah [20]. These sites represent the climatic conditions variety in Saudi Arabia with different solar radiation and wind speed potentials. Yanbu is a major Red Sea port in Al Madinah province of western of Saudi Arabia. It is around 300 km northwest of Jeddah at (24°05′ N 38°00′ E). Dhahran is situated in the eastern part of Saudi Arabia close to the Arabian gulf coast and just a few blocks south of Dammam at (26°16′N 50°09′E). Dhalm is situated in the east of Taif and just about 230 km rounded at (22° 43' 0" N 42° 10' 0" E). Riyadh is the capital and largest city of Saudi Arabia. It is situated in the center of the Arabian Peninsula on a large plateau at (24°38′N 46°43′E). Qaisumah is a village belonging to the city of Hafar Al-Batin, in the eastern province, Saudi Arabia. It is located at around (28°18′35″N 46°7′39″E). A modified wind speed and horizontal solar radiation maps of these sites are shown in Figs 2 and 3, respectively [21].

**Fig 4:**
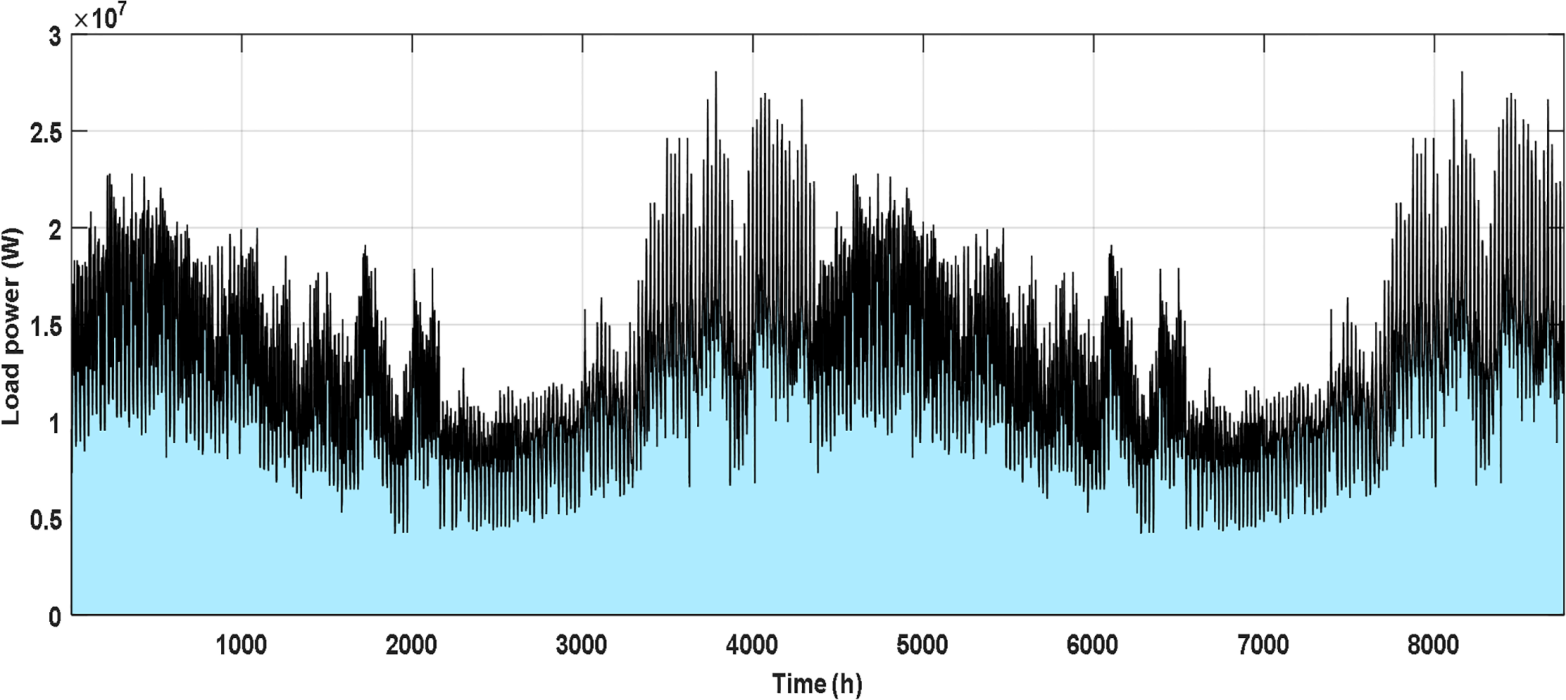
The hourly load demand.

A load demand of Addfa city in Al Jouf province is used for the system under study and has the hourly demand as shown in Fig 4. This load is assumed to be the same on each site of the sites under study. The values of HPL and LPL have been selected based on load survey. The load survey illustrated that it has an agricultural nature and there is a preferred scheduling time slot for the flexible activities (i.e. LPL). Customers feel most convenient if these activities are performed according to their preferences. However a generalized framework has been assumed that if some activity or task is declared as flexible then it can be scheduled in time slots either before or after the preferred time slot for this activity. This paper introduces a detailed design of HRES in case of shifting the load from the low generation periods to high generation periods. This can happen in the real life by increasing the tariff in low generation period and reduce it in high generation period. The response from customers will different depending on many factors. In this paper, for design purpose we assumed 25% of the load can be shifted as a response from customers (LPL), and 75% of the load cannot be shifted and must be supplied whatever the generation conditions (HPL). A sensitivity analysis has been introduced to show the effect of these percentages on the cost of energy generated from the proposed system.

Ten WT from different producers are used in this paper and have the technical characteristics as shown in Table 1 [22].

**Table 1:** The technical characteristics of the WT under study.

WT No.	Manufacturer	*Pr* (kW)	*D* (m)	*u*_*c*_ (m/s)	*u*_*r*_ (m/s)	*u*_*f*_ (m/s)	*h* (m)
WT 1	Enercon-1	330	34	3	13	34	50
WT 2	ACSA-1	225	27	3.5	13.5	25	50
WT 3	Fuhrlander-3	250	50	2.5	15	25	42
WT 4	Ecotecnia-2	600	44	4	14.5	25	45
WT 5	ITP-1	250	30	3	12	25	50
WT 6	NEPC-3	400	31	4	15	25	36
WT 7	Southern Wind Farms	225	29.8	4	15	25	45
WT 8	Enercon-2	330	33.4	3	13	34	37
WT 9	NEPC-2	250	27.6	4	17	25	45
WT 10	India Wind Power	250	29.7	3	15	25	50

### 2.2 Modeling of Wind Energy System

Wind speed at the hub height of WT is calculated by the power law equation using the wind speed data collected at the anemometer height as shown in the following equation [23]:

$$u(h)=u(h_{g})\;{\left(\frac{h}{h_{g}}\right)}^{\alpha }$$ (1)

where, *u*(*h*) and *u*(*h*_*g*_) are wind speeds at hub height (*h*) and anemometer height (*h*_*g*_), respectively, *α* is the roughness factor and has been taken in this paper as 0.14 [23].

The output power of WT is described in terms of wind speed as follows [24]:

$$P_{W}(u)=\left\{ \begin{array}{l} \;0,\;u<u_{c}\;or\;u>u_{f}\\ \;P_{r}\frac{u^{2}-{u_{c}}^{2}}{{u_{r}}^{2}-{u_{c}}^{2}},\;u_{c}\leq u\leq u_{r}\\ \;P_{r},\;u_{r}\leq u\leq u_{f} \end{array}\right.$$ (2)

where, *P*_*W*_ is the WT output power, *P*_*r*_ is the rated output power of WT, *u*_*c*_ is the cut-in wind speed, *u*_*r*_ is the rated wind speed, and *u*_*f*_ is the cut-off wind speed.

The average power generated of each WT in a certain site can be calculated in terms of Weibull parameters and capacity factor as shown in the following equation:

$$P_{WT,av}=C_{F}\times P_{r}$$ (3)

$$C_{F}=\frac{\exp\left[-{\left(u_{C}/c\right)}^{k}\right]-\exp\left[-{\left(u_{r}/c\right)}^{k}\right]}{{\left(u_{r}/c\right)}^{k}-{\left(u_{C}/c\right)}^{k}}-\exp\left[-{\left(u_{f}/c\right)}^{k}\right]$$ (4)

where, *P*_*WT*,*av*_ is WT average power, *C*_*F*_ is the WT capacity factor, *c*, and *k* are the Weibull parameters of WT and can be calculated using the statistical analysis method mentioned in [25].

The average number of WT (*NWT*) required to supply an average annual demand (*P*_*L*,*av*_) can be calculated from the following equation:

$$NWT=\frac{P_{L,}{}_{av}}{P_{WT,av}}$$ (5)

### 2.3 Modeling of PV Energy System

The solar radiation on tilted surface (*H*_*t*_) can be estimated considering the solar insolation, ambient temperature, and manufacturer’s data of the PV panels, slope of the PV panels and latitude and longitude of the site [26]. The output power of the PV array (*P*_*PV*_) is calculated as expressed in the following equation [27]:

$$P_{PV=}H_{t}\times PVA\times \mu_{c}(t)$$ (6)

where, *μ*_*c*_(*t*) is the instantaneous PV cell efficiency, *PVA* is the total solar cells area required to supply the load demand and can be calculated from the following equation:

$$PVA=\frac{1}{24}\sum_{t=1}^{24}\frac{P_{L,av}\left(t\right)F_{s}}{H_{t}\mu_{c}\left(t\right)\eta_{pc}V_{F}}$$ (7)

where, *F*_*s*_ is the safety factor which includes the possible allowance of insolation data inaccuracy, *V*_*F*_ is the factor of variability which considers the impact of yearly radiation variation, their values are around1.1and 0.95, respectively. *η*_*pc*_ is the power conditioning system efficiency [26].

### 2.4 Battery Storage Model

The state of charge (*SOC*) of the battery after certain time (*t*) is calculated based on the energy balance between the wind, PV energy systems and the load as shown in the following equations:

$$E_{B}\left(t+1\right)=E_{B}\left(t\right)\left(1-\sigma \right)+surplus\;power\times \eta_{BC}\;\text{Charging mode}$$ (8)

$$E_{B}\left(t+1\right)=E_{B}\left(t\right)\left(1-\sigma \right)-deficit\;power/\eta_{BD}\;\text{Discharging mode}$$ (9)

where, *E*_*B*_ is the energy of the battery, *η*_*BC*_ and *η*_*BD*_ are the charging and discharging efficiency of the battery (in this paper *η*_*BC*_ and *η*_*BD*_ have been considered as 90% and 85%, respectively) [28]. *σ* is the battery self-discharge rate; it is prescribed to be 0.2% per day for most batteries [29].

### 2.5 Diesel Generator Model

DG is the conventional source of energy which is used as a backup to supply the power deficiency in HRES. The hourly fuel consumption of DG is assessed using the following equation [30]:

$$D_{f}(t)=\alpha_{D}P_{Dg}(t)+\beta_{D}P_{Dgr}$$ (10)

where, *D*_*f*_(*t*) is the hourly fuel consumption of DG in L/h, *P*_*Dg*_ is the average power per hour of the DG, kW, *P*_*Dgr*_ is the DG rated power, kW, *α*_*D*_ and *β*_*D*_ are the coefficients of the fuel consumption curve, L/kWh, these coefficients have been considered as 0.246 and 0.08145, respectively [31].

## 3. A Proposed Power Management Strategy for HRES

The following operational algorithm is proposed for power management of the HRES:

The HPL is supplied primarily from WT and afterward PV array, respectively. In the case of the generated power from the RES surpasses the required power for the HPL (*P*_*LHP*_), the excess power will be used to charge the batteries up to its maximum level (*E*_*B*,*max*_). The abundance power above *E*_*B*,*max*_ will be used to supply the LPL, *P*_*LLP*_. If the power exceeds the LPL demand; the surplus power will be used to supply a typical dummy load (*P*_*dummy*_). Dummy loads, for example, cooling and heating purposes, water pumping and charging batteries of crisis lights. If there is an unmet LPL demand; it will be shifted to the time of surplus generation. This logic is condensed in the following points:

### 3.1 Battery Charge Mode

▪ If *P*_*W*_(*t*) > *P*_*LHP*_(*t*) and *SOC* < *E*_*B*,max_, then;

$$P_{BC}\left(t\right)=\left[\left(P_{W}\left(t\right)-P_{LHP}\left(t\right)\right)\eta_{inv}+P_{PV}\left(t\right)\right]\;\eta_{BC}$$ (11)

▪ If *P*_*W*_(*t*) < *P*_*LHP*_(*t*), [*P*_*W*_(*t*) + (*P*_*PV*_(*t*)*η*_*inv*_)] > *P*_*LHP*_(*t*) and *SOC* < *E*_*B*,max_ then;

$$P_{BC}\left(t\right)=\left[P_{PV}\left(t\right)-\frac{\left(P_{LHP}\left(t\right)-P_{W}\left(t\right)\right)}{\eta_{inv}}\right]\;\eta_{BC}$$ (12)

In all cases of battery charge mode the amassed value of the unmet LPL, *P*_*LLP_sum*_ and *SOC* of the battery can be calculated from the following mathematical statements:

$$P_{LLP\_sum}=P_{LLP\_sum}+P_{LLP}\left(t\right)$$ (13)

$$E_{B}\left(t+1\right)=E_{B}\left(t\right)\left(1-\sigma \right)+P_{BC}\left(t\right)$$ (14)

### 3.2 Feeding the Low Priority Load

▪ If *P*_*W*_(*t*) > *P*_*LHP*_(*t*) and *SOC* ≥ *E*_*B*,max_ then;

$$P_{L\_low}\left(t\right)=\left[\left(P_{W}\left(t\right)-P_{LHP}\left(t\right)\right)+P_{PV}\left(t\right)\eta_{inv}\right]$$ (15)

▪ If *P*_*W*_(*t*) < *P*_*LHP*_(*t*), [*P*_*W*_(*t*) + (*P*_*PV*_(*t*)*η*_*inv*_)] > *P*_*LHP*_(*t*) and *SOC* ≥ *E*_*B*,max_ then;

$$P_{L\_low}\left(t\right)=\left[\left(P_{W}\left(t\right)+\left(P_{PV}\left(t\right)\eta_{inv}\right)\right)-P_{LHP}\left(t\right)\right]$$ (16)

In all cases of feeding LPL, the accumulation of the unmet LPL and *SOC* of the battery can be computed from the following equations:

$$P_{LLP\_sum}=P_{LLP\_sum}+P_{LLP}\left(t\right)-P_{L\_low}\left(t\right)$$ (17)

$$E_{B}\left(t+1\right)=E_{B}\left(t\right)\left(1-\sigma \right)$$ (18)

### 3.3 Feeding the Dummy Load

Dummy load can be used to absorb the surplus renewable generation that exceeds the LPL demand, and battery demand as shown in the following equations:

▪ If *P*_*L_low*_(*t*) > *P*_*LLP_sum*_

$$P_{dummy}\left(t\right)=P_{W}\left(t\right)+P_{PV}\left(t\right)\eta_{inv}-P_{LHP}\left(t\right)-P_{LLP\_sum}$$ (19)

### 3.4 Battery Discharge Mode

If the RES couldn't meet the power required for the HPL, batteries will be utilized to cover the HPL demand until reductions to their minimum level, *E*_*B*,*min*_. The unmet LPL will be shifted to the time of surplus generation. This logic is summarized in the accompanying equations:

▪ If [*P*_*W*_(*t*) + (*P*_*PV*_(*t*)*η*_*inv*_)] < *P*_*LHP*_(*t*) and *SOC* > *E*_*B*,min_ then;

$$P_{BD}\left(t\right)=\frac{\left[P_{LHP}\left(t\right)-P_{W}\left(t\right)-\left(P_{PV}\left(t\right)\eta_{inv}\right)\right]}{\eta_{inv}\;\eta_{BD}}$$ (20)

$$E_{B}\left(t+1\right)=E_{B}\left(t\right)\left(1-\sigma \right)-P_{BD}\left(t\right)$$ (21)

$$P_{LLP\_sum}=P_{LLP\_sum}+P_{LLP}\left(t\right)$$ (22)

where, *P*_*BC*_ and *P*_*BD*_ are charging and discharging power of the battery, respectively, *η*_*inv*_ is the inverter efficiency (in this paper *η*_*inv*_ has been taken as 95% in both directions) [32] and *P*_*L_low*_ is the accessible power used to supply the LPL.

### 3.5 Diesel Generator Operation

If the produced power from HRES is not adequate to supply HPL, the deficit power in HPL will be compensated using the DG as expressed in the following equations:

▪ If [*P*_*W*_(*t*) + (*P*_*PV*_(*t*)*η*_*inv*_)] < *P*_*LHP*_(*t*) and *SOC* ≤ *E*_*B*,min_ then;

$$P_{Dg}\left(t\right)=\left[\left(P_{LHP}\left(t\right)-P_{W}\left(t\right)-\left(P_{PV}\left(t\right)\eta_{inv}\right)\right)\right]$$ (23)

## 4. Problem Statement

The aim of this paper is to introduce an algorithm based on smart grid applications to solve the problem of sizing of HRES to supply the load demand with considering the minimum cost and satisfying a defined reliability index. Demand profile improvement as one of the essential smart grid applications has been covered in this paper. Demand profile improvement helps in smoothing the demand profile, and/or reducing the peak demand or the total energy demand. This will diminish the overall plant and capital cost prerequisites, the cost of the generated energy, and furthermore will increase the system reliability. Demand profile improvement is carried out in this paper by shifting the LPL from low generation to high generation time of RES. In the proposed algorithm, cost estimation is created based on the concept of levelized energy cost (*LEC*) and the reliability of the HRES is produced in view of the concept of loss of load probability (*LOLP*). Cost estimation and reliability assessment of the HRES are detailed in the following subsections:

### 4.1 Reliability Assessment

In the case of the generation from HRES components is insufficient to sustain the HPL and/or LPL, then the load will not get its pressing need and the system will lose its reliability. *LOLP* is viewed as a specialized executed model for the system reliability and can be characterized as [33]:

$$LOLP=\frac{\sum_{0}^{t}Deficit\;Load\;Time}{8760}\;100\%$$ (24)

On account of unmet HPL, the *LOLP* counter will increment when this circumstance happens. The following equations clarify the system operation amid this condition:

▪ If *P*_*W*_(*t*) + *P*_*PV*_(*t*)*η*_*inv*_ + *P*_*Dg*_(*t*) + *P*_*BD*_(*t*) < *P*_*HPL*_(*t*) then;

LOLP_HP=LOLP_HP+1 (25)

where, *LOLP_HP* is the counter for loss of load probability of HPL.

For this situation, the counter *P*_*LLP_sum*_ will increase as given in the mathematical statement (22).

### 4.2 Cost Estimation

*LEC* is a standout amongst the most well-known and utilized indicator of economic analysis of HRES and it can be calculated from the following equation [34]:

$$LEC=\frac{TPV\times CRF}{LAE}$$ (26)

where, *TPV* is the total present cost of the entire system, *LAE* is the annual load demand, and *CRF* is the capital recovery factor. *CRF* and *TPV* are expressed as shown in the following equations:

$$CRF=\frac{r{\left(1+r\right)}^{T}}{{\left(1+r\right)}^{T}-1}$$ (27)

TPV=IC+OMC+RC+FC−PSV (28)

where, *r* is the net interest rate (the interest rate for the genuine monetary condition in Saudi Arabia is 2% [35]), and *T* is the system lifetime in years and *IC* is the initial capital cost of the HRES components, the later can be determined from the following equation:

$$IC=1.4\times PV_{P}\times C_{PV}+1.2\times WT_{P}\times P_{R}\times NWT+E_{BR}\times B_{P}+P_{inv}\times INV_{P}+P_{Dgr}\times DG_{p}$$ (29)

where, *PV*_*P*_ is the PV price per kW ($/kW), *C*_*PV*_ is the rated power of the PV system (kW), *WT*_*P*_ is the WT price per kW ($/kW), *E*_*BR*_ is the battery capacity (kWh), *B*_*P*_ is the battery bank price per kWh ($/kWh), *INV*_*P*_ is the inverter price per kW ($/kW), and *DG*_*P*_ is the DG price per kW ($/kW). *OMC* is the operation and maintenance cost of the HRES segments and can be resolved using the accompanying equations [36]:

$$OMC=OMC_{0}\left(\frac{1+i}{r-i}\right)\;\left(1-{\left(\frac{1+i}{1+r}\right)}^{T}\right)\;r\neq i$$ (30)

$$OMC=OMC_{0}\times T\;r=i$$ (31)

*RC* is the replacement cost of the HRES components and can be determined as shown in the following equation [37]:

$$RC=\sum_{j=1}^{N_{rep}}\left(C_{RC}\times C_{U}\times {\left(\frac{1+i}{1+r}\right)}^{T*j/(N_{rep}+1)}\right)$$ (32)

where, *i* is the inflation rate of replacement units (the inflation rate in Saudi Arabia is 2.3% [38]), *C*_*RC*_ is the capacity of the replacement units, *C*_*U*_ is the cost of replacement units, and *N*_*rep*_ is the number of units replacements over the project lifetime *T*.

*FC* is the DG fuel cost and can be calculated from the following mathematical statement:

$$FC=D_{f}(t)\;DG_{h}\;P_{f}$$ (33)

where, *DG*_*h*_ is the total operation hours of the DG during *T* and *P*_*f*_ is the fuel price per liter ($/L), (fuel price has been considered in this paper as 0.8 $/L).

*PSV* is the present value of scrap and can be expressed in terms of the value of scrap of the system components (*SV*) as shown in the following equation [39]:

$$PSV=\sum_{j=1}^{N_{rep}+1}SV\;{\left(\frac{1+i}{1+r}\right)}^{T*j/(N_{rep}+1)}$$ (34)

The economic and technical parameters of HRES components are shown in Table 2 [40, 41].

**Table 2:** The economic and technical parameters of the HRES components.

Item	Price ($)	***OMC*** *(*%)	***RC*** ($)	***T***	***SV*** (%)	***N***_*rep*_	Salvage times
WT, kW	3000	3	2400	20	20	1	2
Civil work, wind, kW	20%	3	20%	25	20	0	1
PV, kW	2290	1	2000	25	10	0	1
Civil work, PV, kW	40%	1	40%	25	20	0	1
Inverter, kW	711	null	650	10	10	2	3
DG, kW	850	3	850	10	20	2	3
Batteries, kWh	213	3	170	4	20	6	7

## 5. Formulation of the Optimum Sizing Problem

Size estimation of the hybrid PV/wind/battery/diesel energy system is formulated as an optimization problem and the objective function is formulated corresponding to system constraints and performances. The discussion on the objective function and the constraints is detailed in the following subsections:

### 5.1 Objective Function

The objective function of the optimization problem is to minimize the overall system cost *TPV*(*X*). *TPV*(*X*) incorporates capital cost *IC*(*X*), operation and maintenance cost *OMC*(*X*), the replacement cost *RC*(*X*) and the cost of the diesel generator (*DGc*), throughout the lifetime of the installed system. The system lifetime is assumed in this paper to be 25 years. The objective function for optimally designing the HRES must be minimized as expressed by the following equation:

$$\min_{X}\;TPV(X)=\min_{X}\left\{IC(X)+OMC(X)+RC(X)+DGc\right\}$$ (35)

where, *X* is the vector of sizing variables; *X* = *NWT*, *PVA*, *P*_*Dgr*_, and *E*_*BR*_.

### 5.2 Design Constraints

To solve the optimization problem, a set of constraints that must be satisfied with any feasible solution throughout the system operations as the following:

▪ At any time, the *SOC* of the battery bank should satisfy the following constraints:

$$E_{B,\min}\leq E_{B}\left(t\right)\leq E_{B,\max}$$ (36)

$$E_{B}\left(t+1\right)=E_{B}\left(t\right)\left(1-\sigma \right)$$ (37)

▪ At any time, the hourly power generated by DG, *P*_*Dg*_ should be less than or equal the DG rated power, *P*_*Dgr*_ as shown in the following equation:

$$P_{Dg}(t)\leq P_{Dgr}$$ (38)

▪ *LOLP* of the system should be less than allowable *LOLP* reliability index as shown in the following equations:

$$LOLP\_HP<{LOLP\_HP}_{index}$$ (39)

$$P_{LL\_sum}<{P_{LL\_sum}}_{index}$$ (40)

▪ Dummy energy (*E*_*dummy*_) should satisfy the following constraint:

$${E_{dummy}}_{min}<E_{dummy}<{E_{dummy}}_{max}$$ (41)

where, *LOLP_HP*_*index*_ and ${P_{LL\_sum}}_{index}$ are the designed values of the counters *LOLP_HP* and *P*_*LL_sum*_, respectively which are specified by the user. ${E_{dummy}}_{min}$ and ${E_{dummy}}_{max}$ are the minimum and maximum allowable values of the dummy energy and designed by user.

### 5.3 A Proposed Optimization Algorithm for HRES

In this section, a proposed optimization algorithm has been designed to follow the intended values of *LOLP* and *E*_*dummy*_ of the HRES to fulfill an aggregate load demand with minimum *LEC*. In this paper, the value of *LOLP_HP*_*index*_ has been considered to be 4% and ${P_{LL\_sum}}_{index}$ has been taken by (8 days of average LPL, 8*×PL*_*ave_low*_). ${E_{dummy}}_{min}$ and ${E_{dummy}}_{max}$ have been considered to be 0%, 4% of *LAE*, respectively. Along these lines, if (0 < *E*_*dummy*_ < 0.04 × *LAE*), (*P*_*LLP_sum*_ < 8 × 24 × *PL*_*ave_low*_) and (*LOLP_HP* < 0.04), then the optimum size of the HERS components can be obtained. The following step after deciding the optimum size of HERS component is to compute the *LEC*. A PSO algorithm has been utilized as a part of this paper to carry out the optimization problem and decide the optimum problem’s solution.

### 5.4 Implementation of Particle Swarm Optimization Algorithm

**Fig 5:**
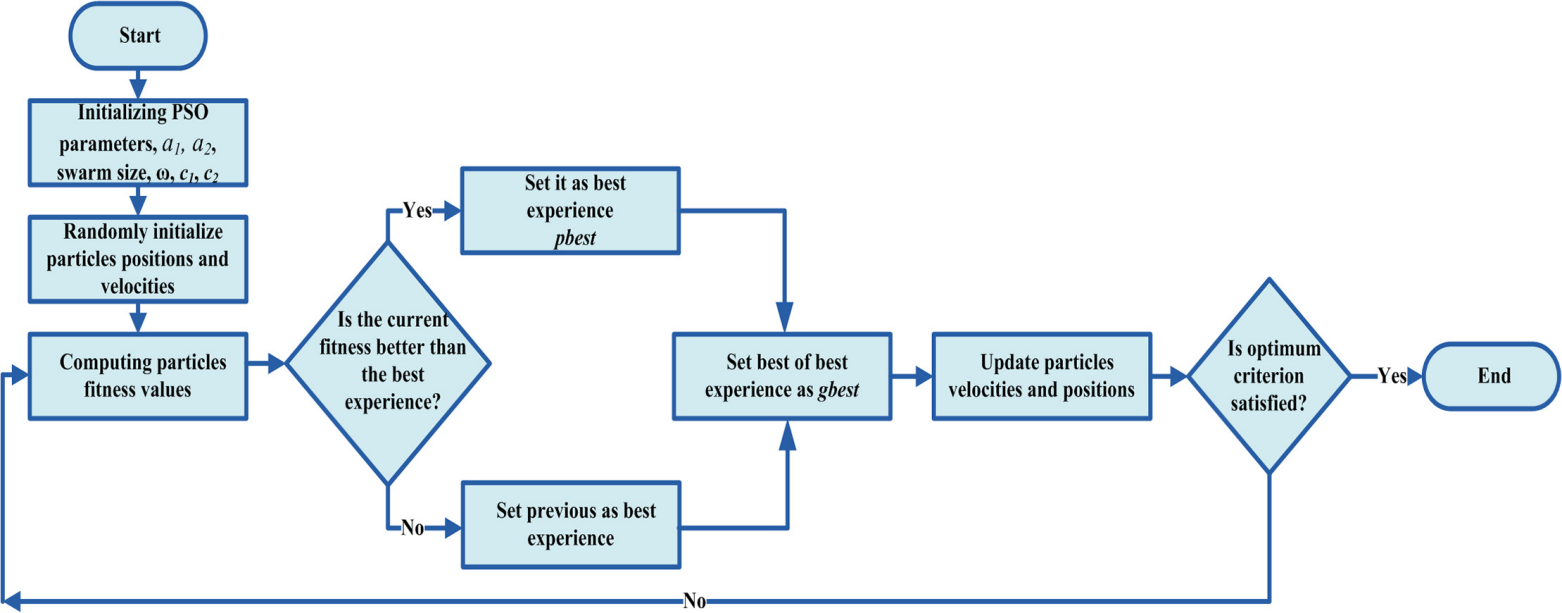
The process of the PSO algorithm.

PSO is a multi-agent parallel search optimization technique, which was presented in 1995 by Kennedy and Eberhart [42]. PSO is an evolutionary technique which is inspired by the social behavior of bird flocking, fish schooling and swarm theory [43, 44]. The PSO idea relies on imposing various particles for searching the optimum solution. Each particle in the PSO algorithm represents a potential solution; these solutions are assessed by the optimization objective function to determine their fitness. In the next iteration, the solutions number doubles until it gets the optimum one. Imposing more particles in each iteration encourage coming to the optimum solution, furthermore decreases the number of optimization iterations. In order to move to the optimum solution, particles move around in a multidimensional search space. The best experience for each particle is stored in the particle memory (*pbest*_*i*_) and the best global obtained among all particles is called as a global best particle (*gbest*). During flight the current position (*x*_*i*_) and velocity (*v*_*i*_) of each particle (*i*) is adapted according to its own experience and the experience of neighboring particles as described by the following equations:

$${v_{i}}^{\left(g+1\right)}=\omega {v_{i}}^{\left(g\right)}+c_{1}a_{1}({pbest}_{i}-{x_{i}}^{\left(g\right)})+c_{2}a_{2}(gbest-{x_{i}}^{\left(g\right)})$$ (42)

$${x_{i}}^{\left(g+1\right)}={x_{i}}^{\left(g\right)}+{v_{i}}^{\left(g+1\right)}$$ (43)

where, g is the counter of generations, and *ω* is the inertia weight factor in a range of [0.5, 1] and almost 1 encourages the global search [45]. *c*_*1*_ and *c*_*2*_ are positive acceleration constants in a range of [0, 4], designated as self-confidence factor and swarm confidence factor, respectively [45], *a*_*1*_ and *a*_*2*_ are uniform randomly generated numbers in a range of [0, 1] [45]. Swarm size, number of particles, *ω*, *c*_*1*_ and *c*_*2*_ are the main parameters of the PSO algorithm, which are initialized by the users, based on the problem being optimized. The process of the PSO algorithm is shown in Fig 5.

To execute the proposed management and optimization procedures of the HRES, a new proposed program based-PSO (NPPBPSO) has been developed. NPPBPSO has been written using MATLAB software in a flexible fashion that is not available in the recent market software such as HySys, HOMER, iHOGA, iGRHYSO, HYBRIDS, RAPSIM, SOMES, HySim, IPSYS, ARES, and SOLSIM [46]. To run NPPBPSO the following information must be accessible:

▪ Initial values of PSO parameters, swarm size, the number of particles, *ω*, *c*_*1*_ and *c*_*2*_.
▪ The optimum design values; *LOLP_HP*_*index*_, ${P_{LL\_sum}}_{index}$, ${E_{dummy}}_{min}$ and ${E_{dummy}}_{max}$.
▪ The geographic data of the sites under study and meteorological data of wind speed, solar radiation, and temperature at these sites.
▪ Specification of WT, PV modules, inverter, batteries, and diesel generator.
▪ The load power data, HPL, and LPL.
▪ Technical and economic data of system components; *T*, *r*, and *i*.

#### 5.4.1 Parallel Implementation of Particle Swarm Optimization

Parallel implementation of particle swarm optimization (PIPSO) can automatically distribute the evaluation of the fitness function and constraints among the ready-made processors or cores. PIPSO is likely to be faster and time saver than the serial implementation of particle swarm optimization (SIPSO); when the fitness function is time-consuming to be computed, or when there is in many particles. Otherwise, the overhead of distributing the evaluation can cause PIPSO to be slower than SIPSO [47]. To use PIPSO a license for parallel computing toolbox software and a parallel worker pool (parpool) must be available.

## 6. Results and Discussion

A PSO-based MATLAB algorithm has been created to determine the optimum size of a PV/WT/batteries/DG system in order to supply a certain load in different remote sites in Saudi Arabia. As indicated in the literature and according to the nature of the problem under study; the suitable values for PSO parameters have been set to make the PSO faster, and exact. The population size has been set to be 20, a maximum number of iterations have been set to be 100, *c*_*1*_ and *c*_*2*_ have been chosen as 2, *a*_*1*_ and *a*_*2*_ have been picked as 0.02, and *ω* has been set as 0.7.

The NPPBPSO gives the possibility to change the penetration ratio (The proportion of wind generation to the total renewable generation (PR)) with certain increment to decide the optimum contribution from HRES. Likewise, the program can chooses the best site out of the sites under study and select the most economic WT for this site.

**Fig 6:**
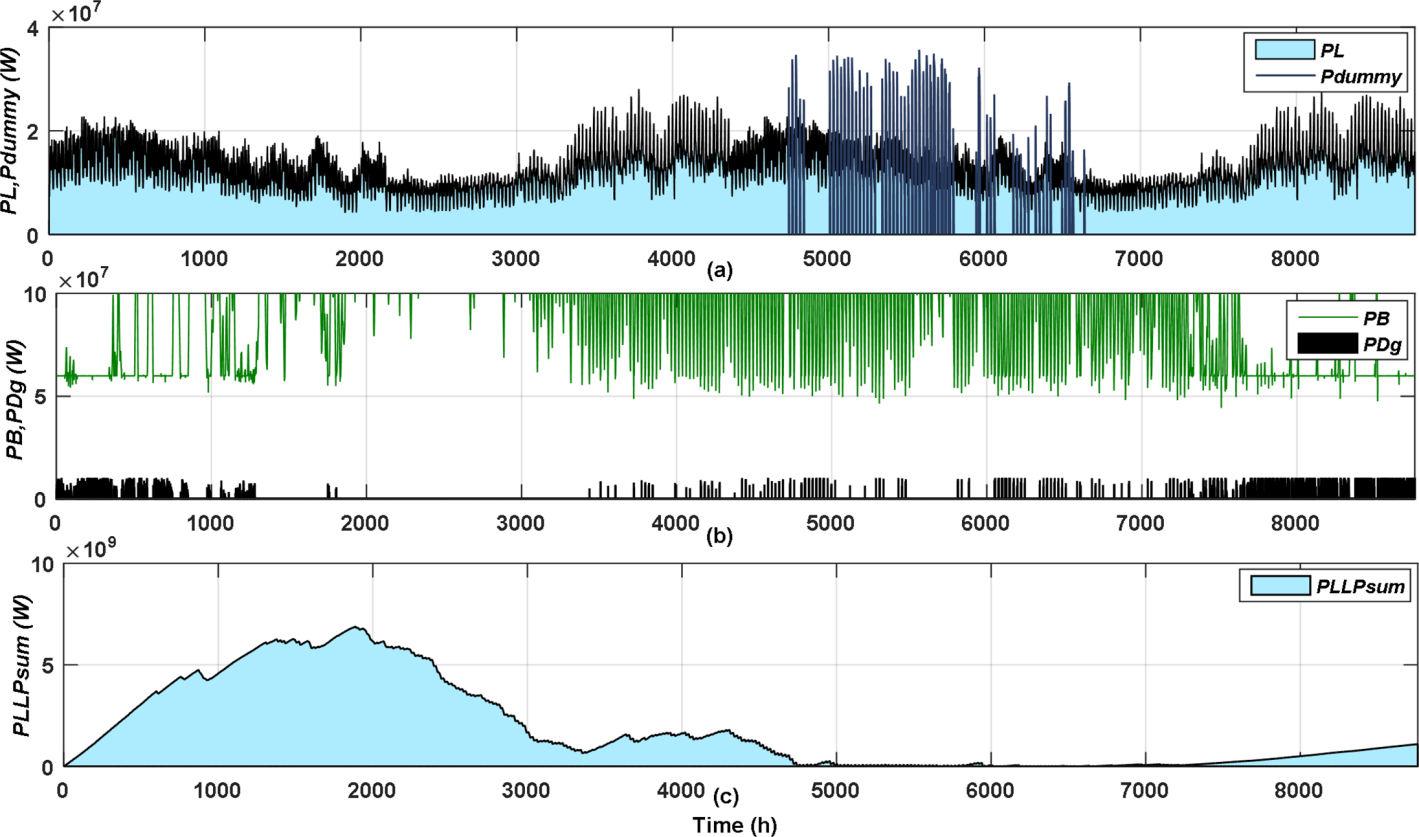
The first optimization iteration of the optimum case.

**Fig 7:**
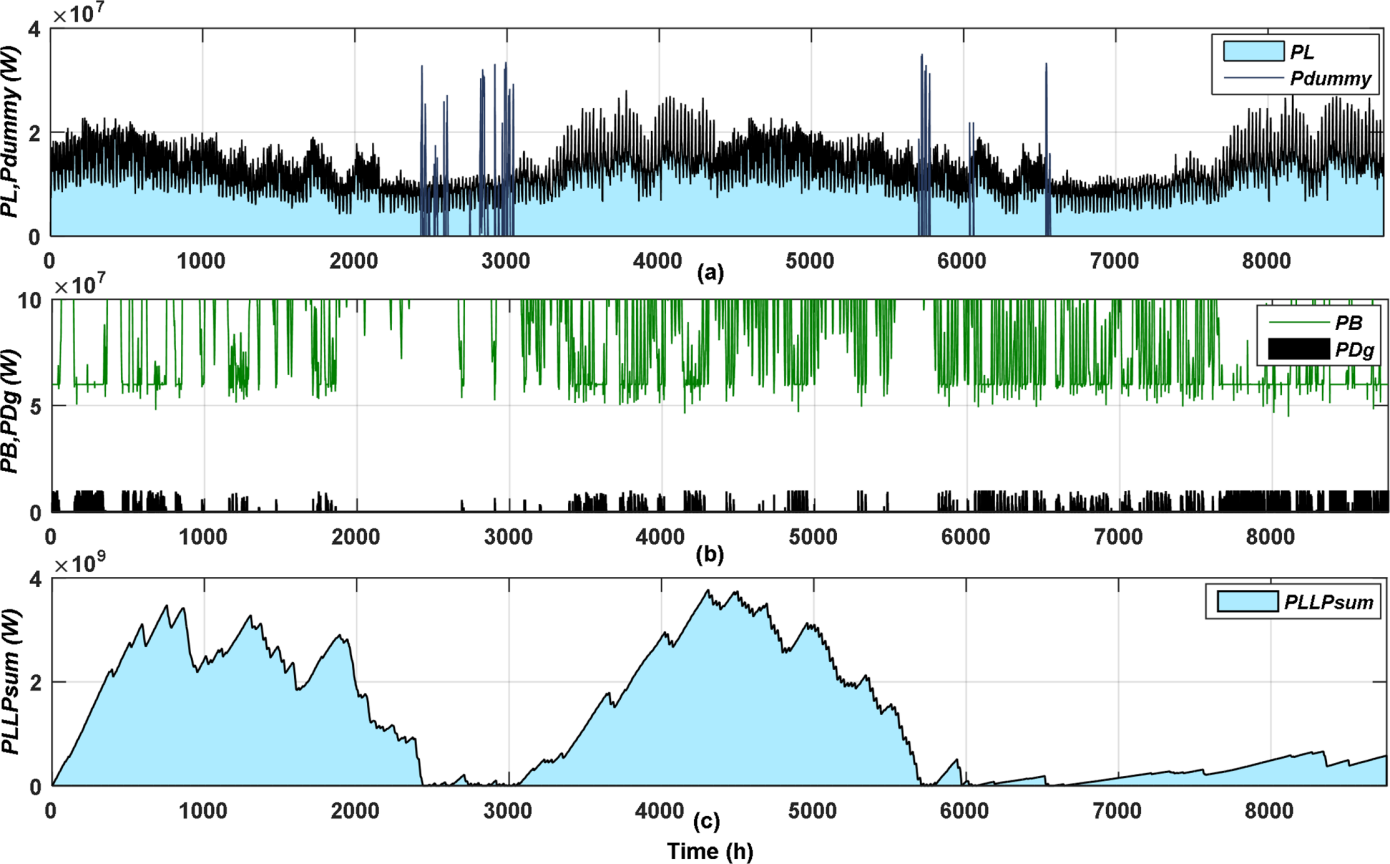
The last optimization iteration of the optimum case.

After initiating the PSO parameters, the PSO algorithm is applied to get the optimum case, the first and last optimization iterations until it gets the optimum design appear in Figs 6 and 7, respectively. The hourly variation of load power (*PL*), dummy power (*P*_*dummy*_) are shown in Figs 6(A) and 7(A), DG power (*P*_*Dg*_), battery accumulated power (*PB*) are shown in Figs 6(B) and 7(B), and the accumulated unmet power of LPL (*P*_*LLP_sum*_) is shown in Fig 6(C) and 7(C). As appeared in Fig 6(A) *E*_*dummy*_ didn’t satisfy the optimum value, therefore, applying NPPBPSO the optimum value can be acquired as is clear from Fig 7(A). The NPPBPSO guarantees to supply the LPL demand as the year progressed, and also can permit low rate of unmet LPL demand to be shifted to the following year, as shown in Figs 6(C) and 7(C). Moreover, the NPPBPSO seeks to distribute the LPL demand through the year using load shifting, to ensure the load not to be concentrated and to reduce the peak load demand, as appeared in Figs 6(C) and 7(C).

The outcomes acquired from NPPBPSO have been compared with those got from IOT. The comparison results are shown in Table 3. As seen in this table, the results obtained from the IOT and NPPBPSO are almost the same. However, there are obvious differences between these two methods summed up in the accuracy and the speed of access the optimum solution as the following:

**Table 3:** The comparison results of IOT and NPPBPSO.

Tools	*c*	*k*	*NWT*	*PVA*	*PSV*	*FC*	*OMC*	*RC*	*LEC*
IOT	5.78	1.97	91	3.80*10^4^	1.80*10^8^	7.18*10^6^	1.2*10^8^	2.9*10^8^	0.2417
NPPBPSO	5.72	1.95	90	3.78*10^4^	1.70*10^8^	6.89*10^6^	1.1*10^8^	2.8*10^8^	0.2334

▪ The solution obtained from PSO is the one satisfy all the optimization constraints and objective function, which thusly make it the exact solution. But, the solution got from IOT is that one satisfies the optimization constraints and objective functions, yet this solution may be away from the optimum solution (global minimum cost). In this way, the most minimal cost acquired with the IOT is not the optimum solution but rather it will be the best plausibility from the accessible solutions.
▪ PSO imposing more particles for each iteration which in turn speed up the access of the optimum solution, but, the IOT impose one solution for each iteration relying upon the experimentation strategy, which in turn may raise the number of iterations until getting the optimum solution. In addition, PSO has the advantage of its ability to solve the complex certifiable problems, high adaptability and ability to manage nonlinearity, non-differentiable functions and functions with an expansive number of parameters. But, the IOT can’t solve variant optimization problems attributable to poorly known objective functions and that have multi-constraints.

**Fig 8:**
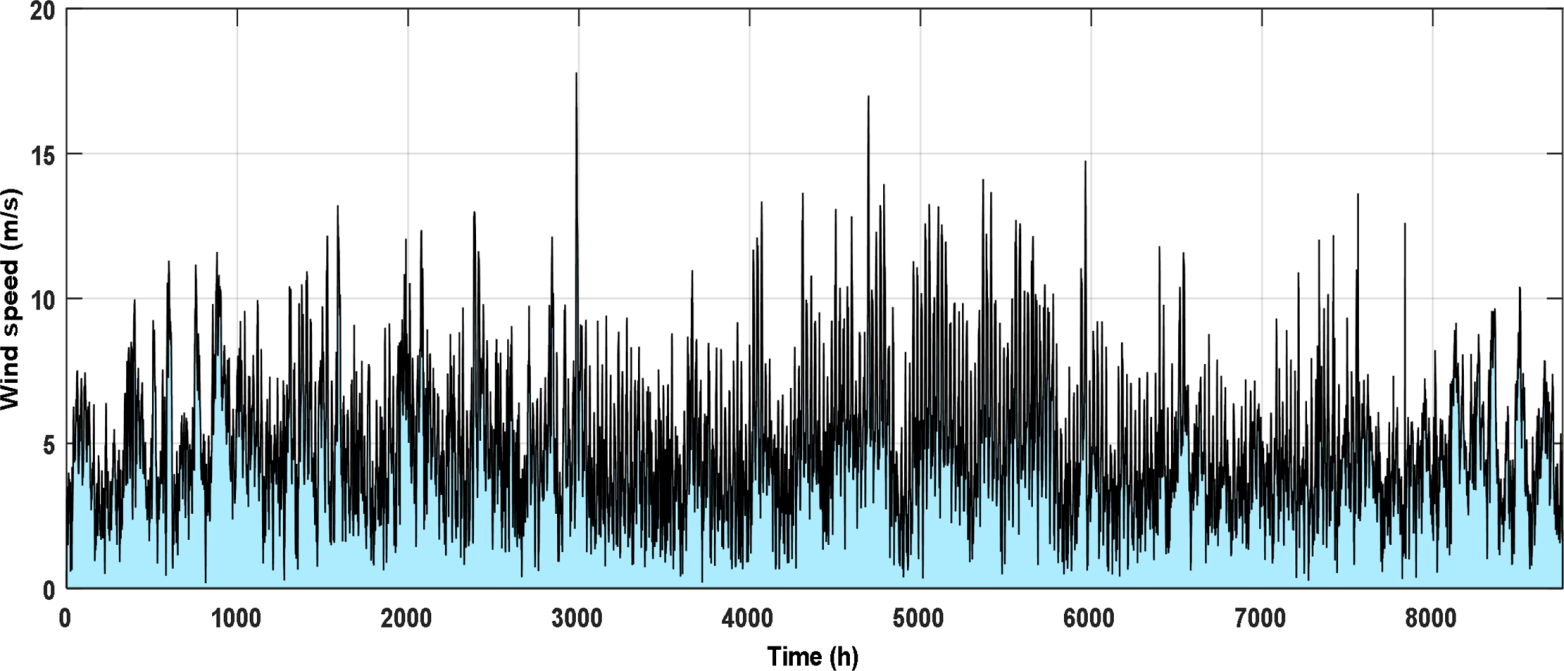
Hourly wind speed for Yanbu site.

**Fig 9:**
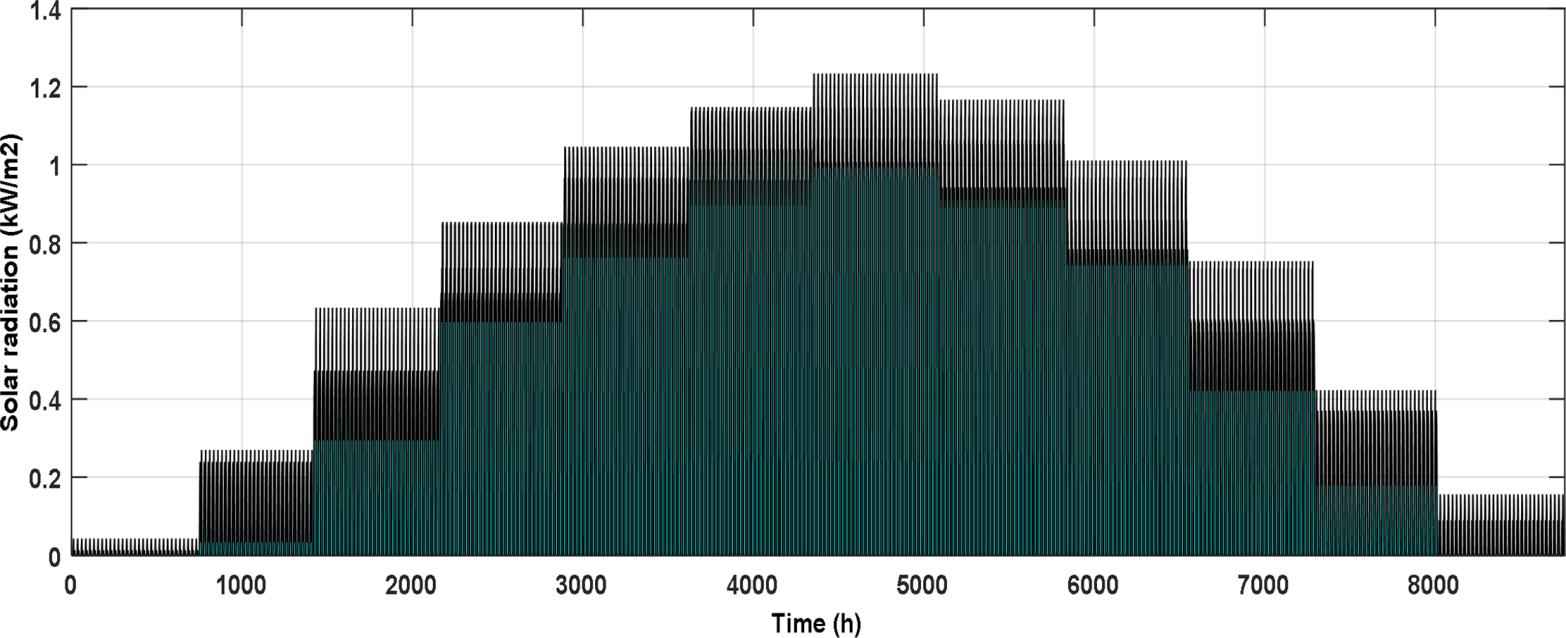
Hourly solar radiation on optimum tilt angle surface for Yanbu site.

The NPPBPSO results affirmed that the minimum *LEC* for the specified limits of *LOLP* and *E*_*dummy*_ was in Yanbu site and the best WT for this site was ITP-1. Additionally, the best share from the RES was at 50% PR. The hourly data of the wind speed at a height of 10 m above the ground level and the insolation on optimum tilt angle surface for Yanbu site appear in Figs 8 and 9, respectively.

**Fig 10:**
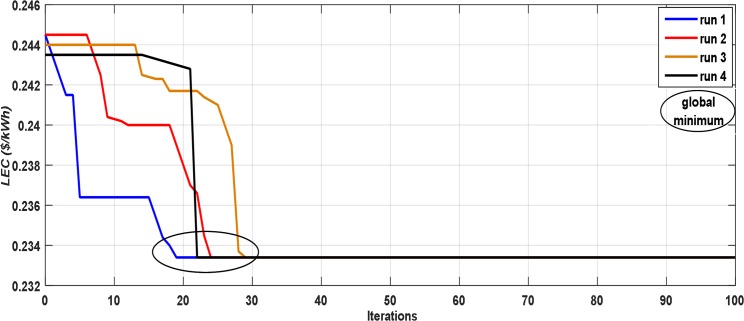
The convergence process of the PSO algorithm for 4 autonomous runs.

**Fig 11:**
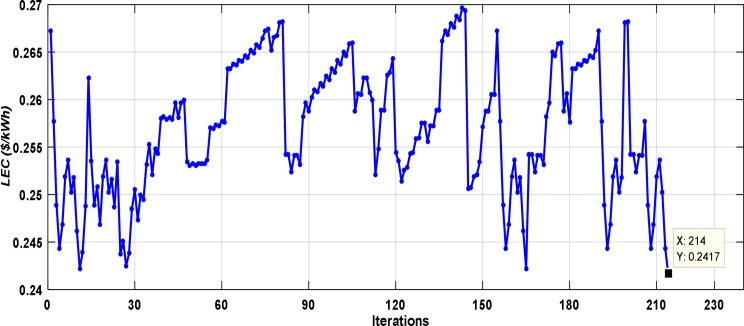
The convergence process of the IOT.

Fig 10 demonstrates the convergence process of the PSO algorithm during the minimization of the *LEC* for 4 autonomous runs. As illustrated in this figure, the optimum solution is acquired after around 30 iterations, and the 100 iterations are considered as a reasonable end measure. In addition, it can be noted that the optimum solution almost converges to the same optimum value (global minimum) for all runs. Fig 11 shows the convergence process for one run of the IOT. As shown in this figure, the optimum solution is obtained after around 215 iterations and this solution not important to be the optimum one. It is also observed that the time taken to find the optimum sizing by using PSO is lower than the one taken by using IOT. Therefore, the optimization utilizing PSO is quicker and more precise than utilizing IOT.

**Fig 12:**
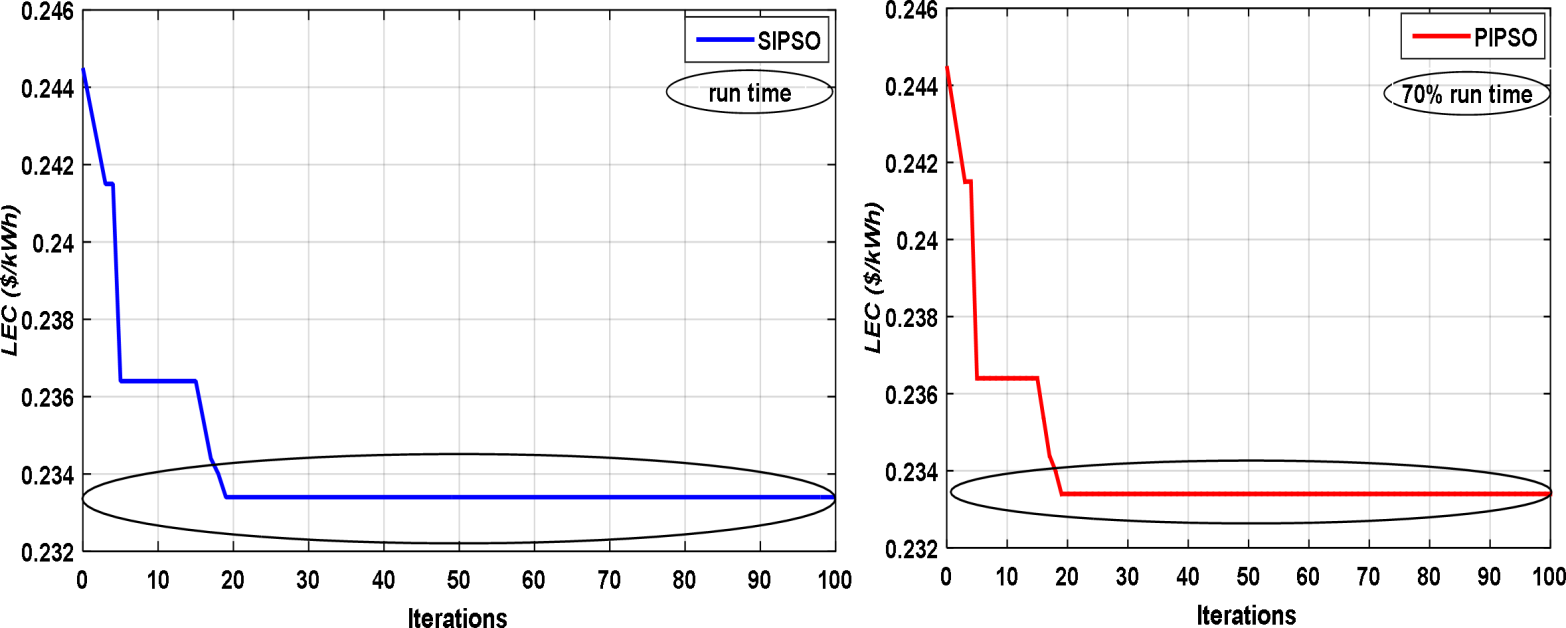
The comparison results of SIPSO and PIPSO.

PIPSO is a granulated approach to speed up the optimization process, to activate PIPSO, parallel choice should set to true. When this condition is true, the NPPBPSO assesses the objective function of the optimization problem in parallel population. Fig 12 shows how to speed up the optimization process by utilizing the PIPSO. Intel® Core™ i5-2410M processor with clock speed: 2.30/2.90 Turbo GHz, 3rd level cache: 3 MB and front side bus: 1333 MHz has been used to run the optimization process. The optimization process has been carried out in a serial manner as appeared in the first part of Fig 12 (SIPSO), and carried out in the second part of Fig 12 using (PIPSO).

As clear from this figure, utilizing the PIPSO can save more time during the optimization process.

**Fig 13:**
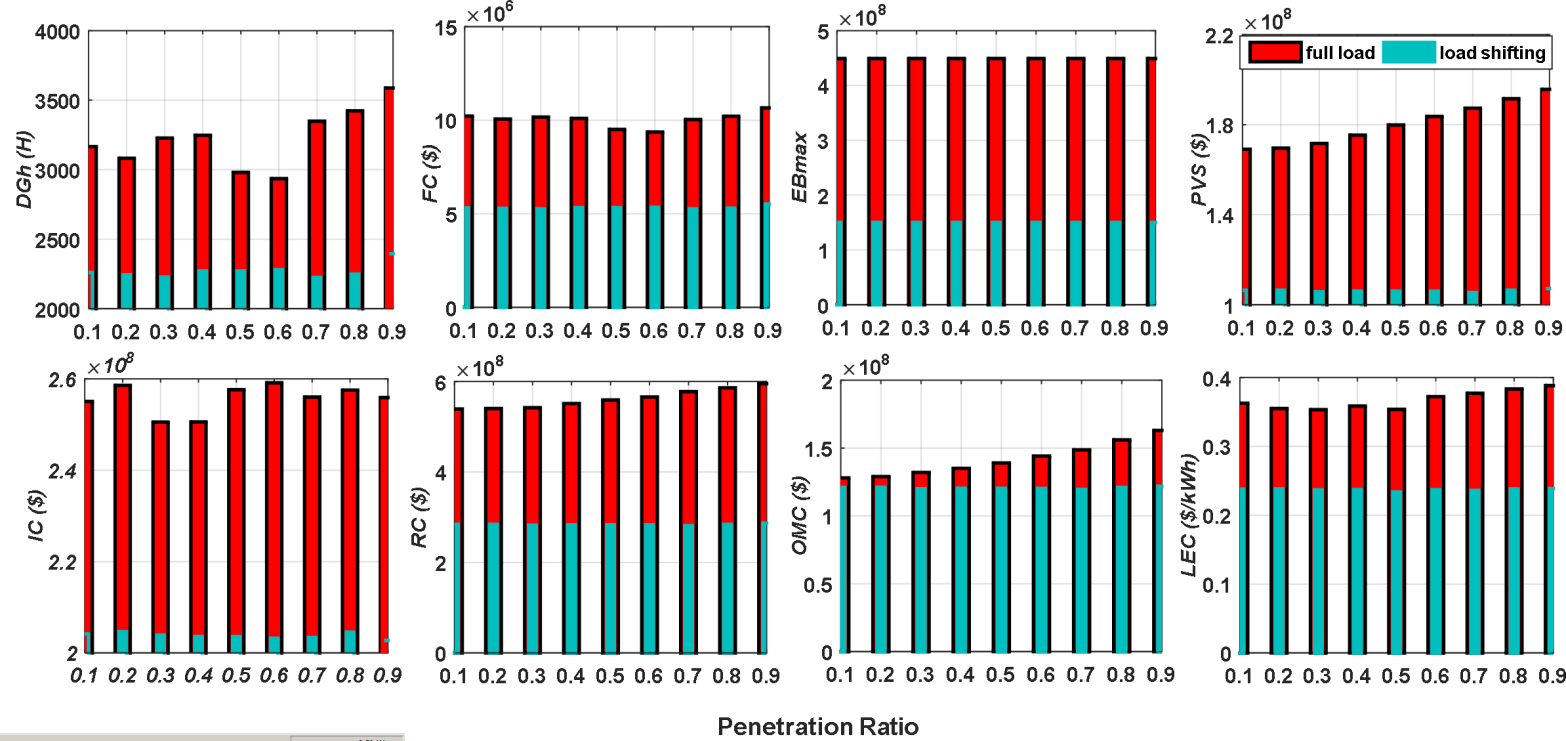
A comparison between full load performance and load shifting performance of HRES with penetration ratio.

The NPPBPSO has been applied to design and optimize the HRES to ensure the load demands in two cases, one for feeding the full load demand and the other one for feeding the load demand with dividing the load into two categories, HPL and LPL. A comparison between these two cases is shown in Fig 13. This figure shows that utilizing load shifting-based load priority reduces the whole system cost, *LEC* and reduces the size of the HRES components, DG capacity, and the battery capacity. Additionally, it lessens the aggregate operation hours of the DG through the system lifetime, and thus diminishes the CO_2_ emission and environment contamination.

**Fig 14:**
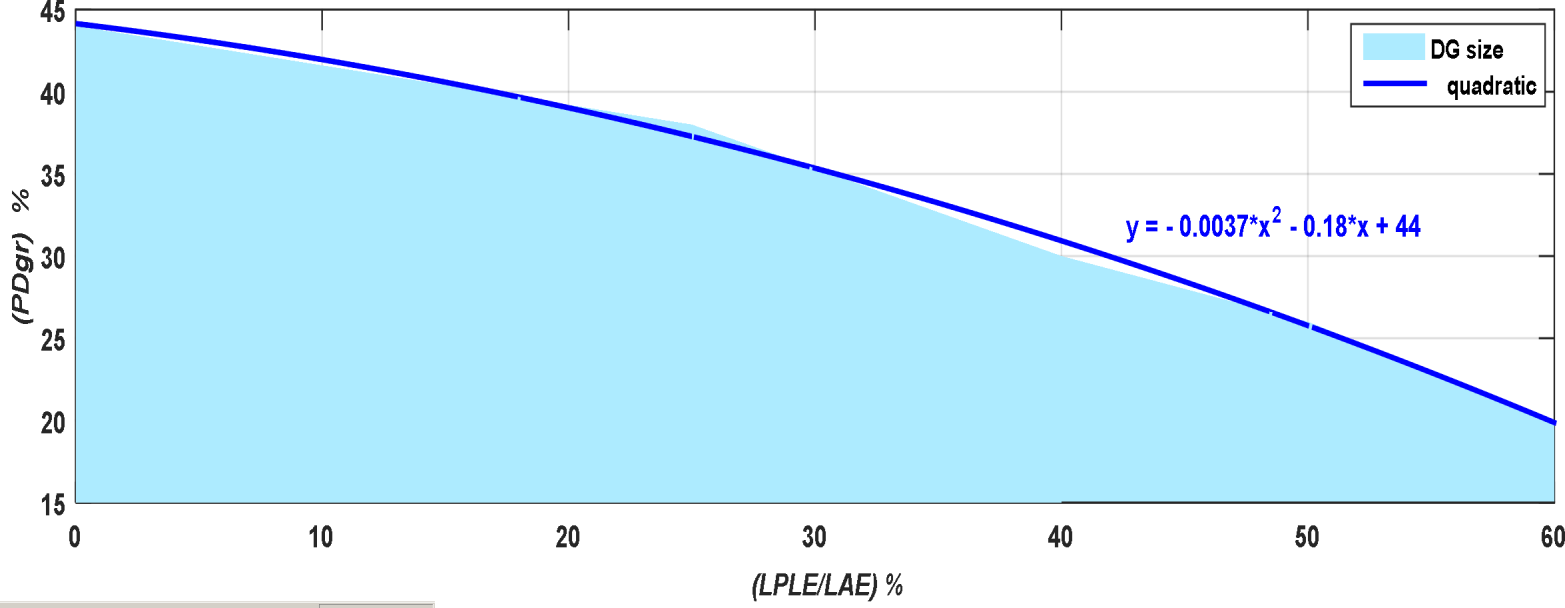
Sensitivity analysis for DG performance with load shifting rate.

Fig 14 describes the sensitivity analysis of the proposed algorithm with the help of quadratic curve fitting. This figure shows the DG performance with relation to the rate of load shifting, at 50% penetration ratio. As shown in this figure, there is an opposite relation between load shifting rate (LPL energy (*LPLE*)*/* annual load energy (*LAE*)) and the DG capacity (*P*_*Dgr*_).

## 7. Conclusions

A methodology for optimum sizing of stand-alone hybrid PV/wind/battery/diesel energy systems utilizing PSO has been presented in this paper. The optimization goal was to minimize the system cost with the state of insuring the load demand and satisfying a set of optimization constraints. Load shifting as one of smart grid applications has been introduced to get a distributed load profile, reduce the entire system cost and reduce CO_2_ emission. Moreover, a methodology to characterize, manage the dummy energy and their exploitation has been presented. Sensitivity analysis has been carried out in this paper to predict the system performance under varying operating conditions. The PSO technique has been implemented in this paper to carry out the optimization process. The simulation results affirmed that PSO is the promising optimization techniques due to its ability to reach the global optimum with relative simplicity and computational proficiency contrasted with the customary optimization techniques. Finally, parallel implementation of PSO has been utilized to speed up the optimization process, and the simulation results confirmed that it can save more time during the optimization process compared to the serial implementation of PSO.
